# The impact of cardiovascular medication use on patients’ daily lives: a cross-sectional study

**DOI:** 10.1007/s11096-018-0601-4

**Published:** 2018-02-12

**Authors:** Danielle M. van der Laan, Petra J. M. Elders, Christel C. L. M. Boons, Giel Nijpels, Janet Krska, Jacqueline G. Hugtenburg

**Affiliations:** 1 Department of Clinical Pharmacology and Pharmacy and the Amsterdam Public Health Research Institute, VU University Medical Center, De Boelelaan 1117, Amsterdam, 1081 HV The Netherlands; 2 Department of General Practice and Elderly Care Medicine and the Amsterdam Public Health Research Institute, VU University Medical Center, Amsterdam, The Netherlands; 3 Medway School of Pharmacy, Universities of Greenwich and Kent, Chatham Maritime, Kent, UK

**Keywords:** Cardiovascular medication, Medication-related burden, Medication non-adherence, Patients’ daily lives, The Netherlands

## Abstract

*Background* The management of multiple long-term medicines of patients with chronic diseases creates a burden for patients. However, limited research is performed on its impact on patients’ daily lives. *Objective* The aim of this study was to explore the impact of cardiovascular medication on different daily life aspects and to examine differences of these aspects between adherent and non-adherent patients. *Setting* Two community pharmacies in the Netherlands. *Method* In this cross-sectional study patients (≥ 45 years) using cardiovascular medication participated. Two equally group sized samples of patients non-adherent as assessed with pharmacy refill data, and patients adherent were selected. *Main outcome measure* Data were collected by means of the Living with Medicines Questionnaire measuring the impact of medicines use on patients’ daily lives. *Results* In total, 196 patients participated, including 96 non-adherent patients. Substantial proportions of patients experienced medication-related burden on different daily life aspects. This burden was mainly related to the acceptance of long-term medicine use, medication-related concerns or dissatisfaction, the interference of medicines with social and daily lives, and the interaction and communication with health care providers. No statistically significant results were found when comparing the impact on patients’ daily lives between adherent and nonadherent patients. *Conclusion* Health care providers should acknowledge the impact of multiple long-term medicines on patient’s daily lives and should make an effort to diminish patients’ medication-related burden by improving patient–provider relationships and by providing adequate treatment information incorporating patients’ individual circumstances. This may facilitate the integration of long-term medicine use in patients’ daily lives.

## Impacts on practice

- Multiple long-term medicines use affects different aspects of patients’ daily lives, and this impact needs to be recognized.
- Health care providers should consider this medication-related burden on patients when managing chronic conditions.
- Health care providers should make an effort to support patients to better integrate long-term medicine use in their daily and social lives.

## Introduction

Patients with chronic diseases are confronted with developing an understanding of the disease and treatment, should attend regular appointments, take several chronic medicines and enact lifestyle changes [1–5]. The management of chronic diseases therefore requires substantial personal investment from patients. Treatment regimens are complex and long-term medicine use creates burden for patients [1]. A systematic review identified five dimensions of medication-related burden: burden related to medication routines, medication characteristics, adverse effects, health care system and social aspects [6]. Excessive medication-related burden may increase the negative impact on patients’ daily lives and negatively affects the health-related quality of life [7, 8]. This is especially the case in patients with cardiovascular disease for whom mostly multiple medicines are prescribed and for whom most medicines prescribed must be used until the end of their life [1, 2]. Patients who experience excessive medication-related burden may encounter problems with adhering to the prescribed regimen [3, 6, 9, 10]. As a consequence, patients become non-adherent to their medication which in its turn leads to increased morbidity and mortality, more hospital admissions and higher health care costs [11, 12]. Limited data on patients’ experienced burden of long-term medicine use and its impact on patients’ daily lives especially in cardiovascular disease are available.

## Aim of the study

The aim of the present study was to explore the impact of chronic cardiovascular medication use on different aspects of patients’ daily lives and to examine the differences of these aspects between adherent and non-adherent patients.

## Ethics approval

All procedures performed in studies involving human participants were in accordance with the ethical standards of the institutional and/or national research committee and with the 1964 Helsinki declaration and its later amendments or comparable ethical standards. The Medical Ethics Committee of the VU University Medical Center Amsterdam approved this study. Informed consent was obtained from all individual participants included in this study.

## Method

### Study design

A cross-sectional study was performed in an urban area of the Netherlands. We intended to include two equally group-sized samples of patients adherent and non-adherent to their prescribed cardiovascular medication. Two community pharmacies participated. Data were collected by means of the Living with Medicines Questionnaire (LMQ-2) [13]. This is an easy-to-use and well-designed instrument to measure the impact of medicine use on patients’ daily lives. The questionnaire has originally been developed in the United Kingdom (UK) based on in-depth interview with patients prescribed four or more regular medicines to explore the issues associated with long-term medicine use [8, 13]. The questionnaire was sent by post to the home addresses of eligible patients.

### Study population

Patients were eligible if they were 45 years or older and were prescribed cardiovascular medication including antihypertensives, antihyperlipidemics and anticoagulants for more than 1 year. Exclusion criteria were patients who were unable to fill out a questionnaire, had insufficient Dutch language skills or used repeat dispensing which is an additionally offered service by the pharmacy.

### Selection procedure

#### Non-adherent sample

The selection method of the Dutch Foundation for Pharmaceutical Statistics (SFK) was used in order to identify non-adherent patients [14]. SFK has been developed by the Royal Dutch Pharmacists Association and collects information on dispensed drugs from the majority of the pharmacies in the Netherlands. Using this software, the Proportion of Days Covered (PDC) was calculated and a list of non-adherent patients (PDC < 80%) was assembled. In each pharmacy a random sample was taken from this list using a randomisation table. A limitation of the SFK method for the selection of non-adherent patients is that data concerning medication refill or medication regimen changes may sometimes be missing. As a result, patients can be falsely classified as non-adherent. Therefore, each patient in the sample was contacted by telephone in order to verify whether the low PDC could be explained by the following factors: (1) visits to another pharmacy to refill medication, (2) hospital admissions, (3) a health care provider initiated discontinuation or (4) changes to the prescribed regimen. Patients able to explain their refill non-adherence with one of the above described aspects in the previous period, were reclassified to the adherent sample. Patients that denied these explanations, were included in the final non-adherent sample.

#### Adherent sample

The pharmacy information and administration system was used to identify adherent patients by making a list of all patients in the pharmacy that met the inclusion criteria. Again a sample of these patients was taken using a randomisation table. In order to only include adherent patients (PDC ≥ 80%), patients that were present on the SFK list as described above were subsequently excluded. The remaining patients in the sample were contacted by telephone and asked for participation.

### Data collection

LMQ-2 measures the impact of medicine use on patients’ daily lives and consists of 42 items divided over eight themes: (1) patient–doctor relationships and communication about medicines, (2) interferences with daily life, (3) practicalities, (4) effectiveness, (5) patient–pharmacist communication about medicines, (6) acceptance of medicine use, (7) autonomy/control over medicine use and (8) concerns about potential harm. Responses are rated on a five-point Likert scale to measure the extent of agreement with the 42 items, ranging from 1 (strongly disagree) to 5 (strongly agree) [15]. The LMQ-2 has been shown to be a valid and reliable multidimensional measure of prescription medicine use experiences and was robust against potential obsequiousness bias [15].

In order to use the LMQ-2 in the Netherlands, the questionnaire was translated using a forward–backward procedure in which the English version was first translated into Dutch by the researchers. The Dutch version was thereafter translated backwards into English by a native speaker in order to verify the accordance with the original English version. The accordance with the English version was verified and approved by the developer of the questionnaire (JK). After the translation process, the feasibility and readability of the Dutch version of the questionnaire was tested in a sample (*n* = 10) of patients using chronic cardiovascular medication using the ‘think-aloud’ method. This method enables to identify difficult or unclear sentences, because patients read aloud every word in each question [16]. The questionnaire was not further adapted based upon the test results.

### Data analysis

Patient characteristics including age, gender, origin, education level, employment status, living situation, assistance with medication use from others and number of prescribed medicines were obtained. Means and standard deviations for continuous variables and frequencies and percentages for categorical variables were calculated. The 42 items were both positively and negatively phrased. Reverse scoring enabled uniformity in the direction of responses, with a higher score indicating more impact of medicines use on patients’ daily lives. The LMQ-2 sum score was obtained by summing the scores for each item and was presented as means and standard deviations. The sum score ranges from 42 to 210. In addition, a theme sum score was calculated for each of the eight themes. Independent samples *t* tests were used to compare sum and theme scores between the adherent and non-adherent sample. When examining the LMQ-2 scores on item level, the scale was dichotomised into 1 (strongly agree, agree with item) and 0 (strongly disagree, disagree, neutral with item) [17]. Proportions of patients agreeing with the LMQ-2 items were presented as frequencies and percentages for the total study population. Furthermore, logistic regression analyses were used in order to examine the differences in LMQ-2 item scores between adherent and non-adherent patients. Odds ratios (OR) with 95% confidence intervals (CI) and *p* values were presented. A *p* value of ≤ 0.01 was considered statistically significant. Statistical analyses were performed using SPSS version 22.0 (IBM Corp, Armonk, NY, USA).

## Results

### Selected patients

**Fig. 1:**
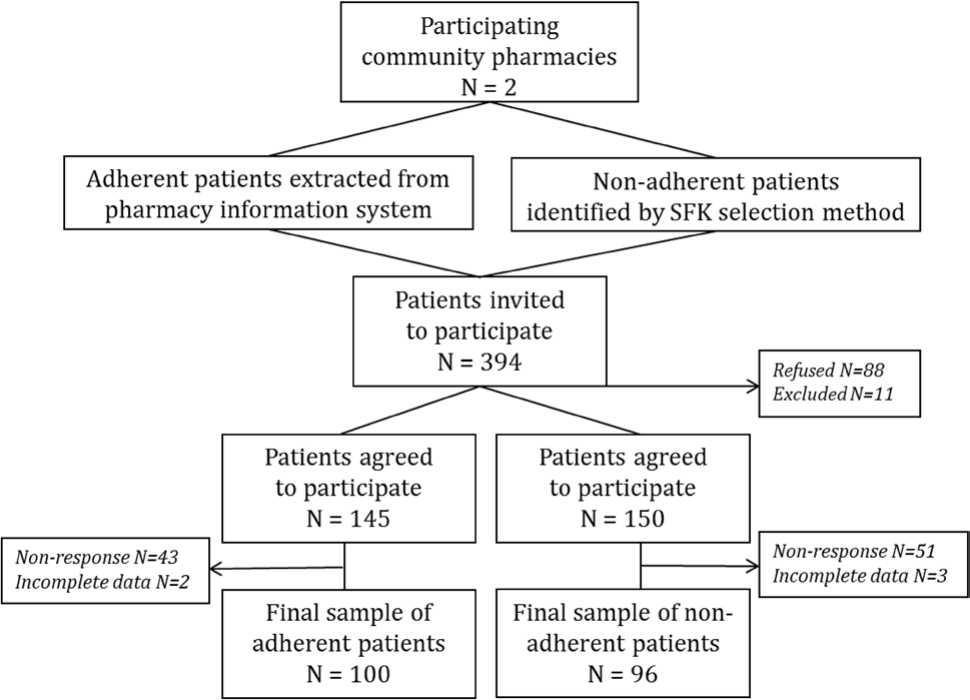
Flow diagram of study population

A total of 394 patients were invited to participate (Fig. 1**)**. Of patients willing to participate (*n* = 295), 94 patients did not respond to the questionnaire and five individuals were excluded due to missing questionnaire data. The final sample consisted of 196 patients, including 100 and 96 patients in the adherent and non-adherent sample, respectively.

### Patient characteristics

In Table 1 the patient characteristics are listed. In the total study population the mean age was 71.0 years and 51.0% was male. Patients were predominantly from Dutch origin (89.8%), retired (65.8%) and lived with others (74.0%). About two-thirds of patients was prescribed at least four medicines. No significant differences in patient characteristics between the adherent and non-adherent sample were found, except for number of prescribed medicines. Significantly more non-adherent patients used more than four prescribed medicines (*p* = 0.004) than adherent patients.

**Table 1:** Characteristics of the study population

Patient characteristics	Total study population (N = 196)	Adherent sample (N = 100)	Non-adherent sample (N = 96)
	N (%) or mean ± SD	N (%) or mean ± SD	N (%) or mean ± SD
Age (years)	71.0 ± 10.6	71.4 ± 10.2	70.5 ± 11.0
*Gender*			
Male	100 (51.0)	54 (54.0)	46 (47.9)
*Origin*			
Dutch	176 (89.8)	93 (93.0)	83 (86.5)
*Education*			
Low	47 (24.0)	21 (21.0)	26 (27.1)
Moderate	56 (28.6)	29 (29.0)	27 (28.1)
High	93 (47.4)	50 (50.0)	43 (44.8)
*Employment status* ^a^			
Employed	53 (21.9)	23 (23.0)	20 (20.8)
Volunteer	15 (7.7)	7 (7.0)	8 (8.3)
Retired	129 (65.8)	66 (66.0)	63 (65.6)
Unemployed/disabled	19 (9.7)	9 (9.0)	10 (10.4)
*Living situation*			
With others	145 (74.0)	75 (75.0)	70 (72.9)
*Assistance with medication use*			
Yes	22 (11.2)	12 (12.0)	10 (10.4)
*No. of prescribed medicines* ^b,c^			
≥ 4	133 (67.9)	59 (60.2)	74 (79.6)

*SD* standard deviation

^a^Total exceeds 100%

^b^Data from five patients is missing

^c^Significantly different between adherent and non-adherent patients (*p* = 0.004)

### Impact of medicines use on daily life

In Table 2 the results of the LMQ-2 sum scores and themes scores are presented. In the total study population the mean sum score was 93.1 (SD 13.6). For the adherent and non-adherent patients, the sum score was 93.2 (SD 13.3) and 93.0 (SD 14.0), respectively. No statistically significant differences between the adherent and non-adherent patients were found for neither the LMQ-2 sum score nor LMQ-2 theme scores.

**Table 2:** LMQ-2 sum scores and theme scores of adherent and non-adherent patients

	Total study population (N = 196)	Adherent patients (N = 100)	Non-adherent patients (N = 96)	*p* value^a^
	Mean ± SD	Mean ± SD	Mean ± SD	
Sum score (range 42–210)	93.1 ± 13.6	93.2 ± 13.3	93.0 ± 14.0	0.931
*Theme scores*				
1 Patient–doctor relationships and communication about medicines (9–45)	19.8 ± 4.6	19.8 ± 4.0	19.8 ± 5.2	0.985
2 Interferences with daily life (8–40)	17.7 ± 4.4	17.7 ± 4.5	17.8 ± 4.3	0.909
3 Practicalities (7–35)	13.3 ± 3.7	13.3 ± 3.8	13.3 ± 3.6	0.989
4 Effectiveness (4–20)	8.9 ± 2.3	9.0 ± 2.2	8.9 ± 2.4	0.743
5 Patient–pharmacist communication about medicines (3–15)	6.2 ± 1.8	6.2 ± 1.7	6.2 ± 1.8	0.848
6 Acceptance of medicine use (4–20)	8.3 ± 2.7	8.3 ± 2.5	8.3 ± 2.9	0.960
7 Autonomy/control over medicine use (4–20)	12.8 ± 2.8	13.1 ± 2.9	12.5 ± 2.8	0.157
8 Concerns about potential harm (3–15)	8.4 ± 2.7	8.1 ± 2.8	8.6 ± 2.6	0.179

*LMQ* Living with Medicines Questionnaire, *SD* standard deviation

^a^Independent samples *t* tests

In Table 3 the results of the LMQ-2 scores on item level are presented. A selection of notable results on the proportions of patients agreeing with the items are described below. In both the adherent and non-adherent sample almost a quarter of patients indicated not to trust the doctor in choosing their medicines (24.0%) and almost half of the patients indicated that their doctor does not always take their concerns of side effects seriously (42.3%). About 40% of patients indicated they were concerned about experiencing side effects or were concerned about long-term effects of taking medicines. For up to 35% of patients medicines adversely affected their social and daily lives, including not living their life as they want to (34.7%) and experiencing interference with their social life (23.0%). A majority of the patients indicated not to be able to adapt their medicine-taking to their lifestyles (57.7%). One out of four patients indicated they did not accept that they have to take medicines long term (23.0%). Over one-third of patients (35.2%) indicated not being confident about speaking with the pharmacist about medicines.

**Table 3:** Differences in proportions of patients agreeing with 42 LMQ items between adherent and non-adherent patients

	Total study population (N = 196)	Adherent sample (N = 100)	Non-adherent sample (N = 96)	Odds ratio (95% CI)	*p* value^a^
	Agree or strongly agree N (%)	Agree or strongly agree N (%)	Agree or strongly agree N (%)		
*Theme 1 Patient*–*doctor relationships and communication about medicines*					
My doctor(s) listens to my opinions and concerns about my medicines	142 (72.4)	74 (74.0)	68 (70.8)	0.85 (0.46–1.60)	0.620
The information my doctor(s) gives me about my medicines is useful	170 (86.7)	88 (88.0)	82 (85.4)	0.80 (0.35–1.83)	0.595
My doctor(s) spends enough time discussing my medicines with me	146 (74.5)	78 (78.0)	68 (70.8)	0.69 (0.36–1.31)	0.251
I am confident speaking to my doctor(s) about my medicines	145 (74.0)	76 (76.0)	69 (71.9)	0.81 (0.43–1.53)	0.511
My doctor(s) takes my concerns of side effects seriously	113 (57.7)	56 (56.0)	57 (59.4)	1.15 (0.65–2.03)	0.633
I understand what my doctor(s) tells me about my medicines	169 (86.2)	87 (87.0)	82 (85.4)	0.88 (0.39–1.97)	0.748
The health professionals providing my care know enough about me and my medicines	125 (63.8)	65 (65.0)	60 (62.5)	0.90 (0.50–1.61)	0.716
I trust the judgement of my doctor(s) in choosing medicines for me	149 (76.0)	74 (74.0)	75 (78.1)	1.26 (0.65–2.43)	0.499
There is enough sharing of information about my medicines between professionals providing my care	86 (43.9)	43 (43.0)	43 (44.8)	1.08 (0.61–1.89)	0.801
*Theme 2 Interferences with daily life*					
Taking medicines interferes with my social life	45 (23.0)	26 (26.0)	19 (19.8)	0.70 (0.36–1.38)	0.303
Taking medicines causes problems with daily tasks	17 (8.7)	6 (6.0)	11 (11.5)	2.03 (0.72–5.72)	0.182
The medicines I use have an adverse effect on the holidays I can take	8 (4.1)	6 (6.0)	2 (2.1)	0.33 (0.07–1.69)	0.185
My life revolves around using my medicines	55 (28.1)	27 (27.0)	28 (29.2)	1.11 (0.60–2.08)	0.736
Taking medicines affects my driving ability	8 (4.1)	5 (5.0)	3 (3.1)	0.61 (0.14–2.64)	0.511
I have to put a lot of planning and thought into taking my medicines	10 (5.1)	3 (3.0)	7 (7.3)	2.54 (0.634–0.14)	0.186
I worry that I have to take several medicines at the same time	16 (8.2)	8 (8.0)	8 (8.3)	1.05 (0.38–2.91)	0.932
Changes in daily routine cause problems with my medicines	41 (20.9)	23 (23.0)	18 (18.8)	0.77 (0.39–1.54)	0.465
*Theme 3 Practicalities*					
It is difficult to identify which medicine is which	20 (10.2)	11 (11.0)	9 (9.4)	0.84 (0.33–2.12)	0.707
The instructions on my medicines are easy to follow	187 (95.4)	94 (94.0)	93 (96.9)	1.98 (0.48–8.15)	0.345
I find opening the packaging of my medicines difficult	28 (14.3)	17 (17.0)	11 (11.5)	0.63 (0.28–1.43)	0.270
I find getting my prescriptions from the doctor difficult	14 (7.1)	6 (6.0)	8 (8.3)	1.42 (0.48–4.27)	0.528
I find getting my medicines from the pharmacist difficult	11 (5.6)	6 (6.0)	5 (5.2)	0.86 (0.25–2.92)	0.810
I find using my medicines difficult	21 (10.7)	5 (5.0)	16 (16.7)	3.80 (1.33–10.83)	0.012*
It is easy to keep to my medicines routine	171 (87.2)	89 (89.0)	82 (85.4)	0.72 (0.31–1.68)	0.454
*Theme 4 Effectiveness*					
I am satisfied with the effectiveness of my medicines	131 (66.8)	67 (67.0)	64 (66.7)	0.99 (0.54–1.79)	0.960
My medicines live up to my expectations	131 (66.8)	67 (67.0)	64 (66.7)	0.99 (0.54–1.79)	0.960
My medicines are working	157 (80.1)	82 (82.0)	75 (78.1)	0.78 (0.39–1.58)	0.498
My medicines prevent my condition getting worse	126 (64.3)	60 (60.0)	66 (68.8)	1.47 (0.81–2.64)	0.202
*Theme 5 Patient*–*pharmacist communication about medicines*					
The information my pharmacist gives me about my medicines is useful	162 (82.7)	82 (82.0)	80 (83.3)	1.10 (0.52–2.30)	0.805
I am confident speaking to my pharmacist about my medicines	127 (64.8)	65 (65.0)	62 (64.6)	0.98 (0.55–1.77)	0.951
I understand what my pharmacist tells me about my medicines	166 (84.7)	81 (81.0)	85 (88.5)	1.81 (0.81–4.04)	0.146
*Theme 6 Acceptance of medicine use*					
Taking medicines is routine for me	146 (74.5)	73 (73.0)	73 (76.0)	1.17 (0.62–2.24)	0.625
I accept that I have to take medicines long term	151 (77.0)	76 (76.0)	75 (78.1)	1.13 (0.58–2.20)	0.724
My medicines are important to me	179 (91.3)	90 (90.0)	89 (92.7)	1.41 (0.52–3.88)	0.502
My medicines allow me to live my life as I want to	128 (65.3)	72 (72.0)	56 (58.3)	0.54 (0.30–0.99)	0.046*
*Theme 7 Autonomy/control over medicine use*					
I can vary the dose of the medicines I take	19 (9.7)	9 (9.0)	10 (10.4)	1.18 (0.46–3.03)	0.738
I can change the times I take my medicines if I want to	101 (51.5)	44 (44.0)	57 (59.4)	1.86 (1.06–3.28)	0.032*
I can choose whether or not to take my medicines	67 (34.2)	29 (29.0)	38 (39.6)	1.60 (0.89–2.91)	0.120
I can adapt my medicine-taking to my lifestyle	83 (42.3)	43 (43.0)	40 (41.7)	0.95 (0.54–1.67)	0.856
*Theme 8 Concerns about potential harm*					
I am concerned about experiencing side effects	79 (40.3)	34 (34.0)	45 (46.9)	1.71 (0.96–3.05)	0.067
I am concerned about possible damaging long-term effects of taking medicines	78 (39.8)	36 (36.0)	42 (43.8)	1.38 (0.78–2.46)	0.286
I worry that my medicines may interact with each other	20 (10.2)	10 (10.0)	10 (10.4)	1.05 (0.42–2.64)	0.923

*CI* confidence interval, *LMQ* Living with Medicines Questionnaire

^a^Logistic regression analyses *p ≤ 0.05; **p ≤ 0.01 (= statistical significant)

When comparing the adherent and non-adherent patients, no statistically significant differences between groups were found on LMQ-2 item level. For one item, ‘I find using my medicines difficult’ (*p* = 0.012), the proportion of non-adherent patients was marginally significantly higher than adherent patients. Other trends were that a lower proportion of non-adherent patients agreed with the statement: ‘My medicines allow me to live my life as I want to’ (*p* = 0.046), and a higher proportion with the statement: ‘I can change the times I take my medicines if I want to’ (*p* = 0.032).

## Discussion

This study demonstrated that the experienced burden of cardiovascular medication use on patients’ daily lives was mainly related to the acceptance of long-term medicine use, medication-related concerns or dissatisfaction, the interference of medicines with social and daily lives, and the interaction and communication with health care providers. There were no significant differences in experienced burden between the adherent and non-adherent sample.

In this study, substantial proportions of patients experienced medication-related burden. The majority of these findings resonate with the results of two other studies administrating the LMQ [17, 18]. These studies concluded that long-term use of medicines was burdensome and may negatively affect patients’ quality of life. The finding of our study that the experienced medication-related burden was related to different daily life aspects, corresponds with other literature. In a review of Sav et al. [19] different dimensions of treatment burden were identified in multiple studies including side effects of treatment, the economic burden imposed by treatment, time required to obtain, administer and manage treatment, and the psychosocial aspects of burden including the impact on social and daily lives. In contrast to this review, no conclusions can be drawn about the experienced economic burden in our study population since no costs related aspects were assessed in the questionnaire. In a review of Rosbach and Andersen [20] it was also concluded that the burden of treatment is a complex concept consisting of many different components and factors interacting with each other. They also found that patients seem to use strategies to diminish the burden and try to routinize and integrate complex treatment into their daily lives. Since, the experienced medication-related burden is also related to the interaction with health care providers, a structural change in health care delivery is required to diminish patients’ burden [21]. Therefore, it is important for health care providers to recognise that poor patient–provider relationships may lead to increased burden and that they should make an effort to improve communication about patients’ attitudes and concerns, involve patients in treatment decisions and incorporating individual’s circumstances and preferences [1, 4, 7]. This may increase the chance to better integrate long-term treatment in patients’ daily lives. This finding was also confirmed by a review of Mohammed et al., which indicated a need for health care providers to have more insight into patients’ medication-related burden since it plays a central role in influencing beliefs and behaviour towards medicines. By understanding patients’ experienced burden, health care providers can provide individualised care and assist patients in improving medication therapy and health outcomes [6].

No statistically significant results were found when comparing the burden on patients’ daily lives between the adherent and non-adherent sample. The findings did not support our original hypothesis that non-adherent patients might perceive more medication-related burden than adherent patients. The similar level of burden found between these two groups is therefore interesting and requires further exploration. The fact that the questionnaire was not able to distinguish between adherent and non-adherent patients can have several explanations. First, the perceived burden of chronic medication in adherent and non-adherent patients might be similar. Second, adherent and non-adherent patients may cope in a differential manner with this burden. A questionnaire that is specifically designed to measure burden and not the coping mechanisms to manage this burden is not suitable to identify these differences. In addition, rather than in the experienced burden, the beliefs about medicines might influence medication intake behaviour [22, 23]. The marginal differences between adherent and non-adherent patients in a few LMQ-2 items, including finding medicines use difficult, feeling in control of changing times of medicine intake, and feeling that medicines allow living life as wanted, could be explained to support in this direction. However, these differences should be viewed with caution due to multiple testing. It might be interesting to elaborate on the possible relationship of these items with non-adherence in further research.

### Limitations

Some limitations need to be discussed. First, the accuracy of the selection method of SFK to identify non-adherent patients is limited. It may occur that certain data in SFK is missing whereupon it is possible to falsely classify patients as non-adherent. However, to minimise bias missing SFK data was verified with each patient and when needed a patient was reclassified. Another limitation was that the sample size was maybe not large enough to find differences between adherent and non-adherent patients. A final limitation was that the adherent and non-adherent samples were slightly different on patient characteristics. The samples differed on the number of prescribed medicines. In the UK, LMQ-2 scores have been shown to be related to the number of prescribed medicines [15]. However, in our sample no significant correlation was found (data not shown), but again this may be due to insufficient sample size.

## Conclusion

This study demonstrated that substantial proportions of patients using chronic cardiovascular medication experienced medication-related burden on different daily life aspects. Health care providers must acknowledge the impact of multiple long-term medicine use on patients’ daily lives and should make an effort to diminish patients’ medication-related burden. Therefore, patient–provider relationships and their communication need to be improved, incorporating patients’ individual circumstances and preferences in order to facilitate the integration of long-term medicine use in patients’ daily lives. We did not find differences in experienced burden between adherent and non-adherent patients. It shows that we might underestimate the burden in adherent patients, which is an interesting finding. Further research could explore this and the potential effects of intervention strategies aimed at coping mechanisms for medication-related burden on patients’ medication adherence.
